# Supplementary material for: Propulsive Force Modulation Drives Split-Belt Treadmill Adaptation in People with Multiple Sclerosis
Source: Sensors (Basel). 2024 Feb 6;24(4):1067. doi: 10.3390/s24041067 (PMC10891828; doi:10.3390/s24041067)
Supplement: Supplementary file 1 [file sensors-24-01067-s001.zip › sensors-2840132-supplementary.pdf]

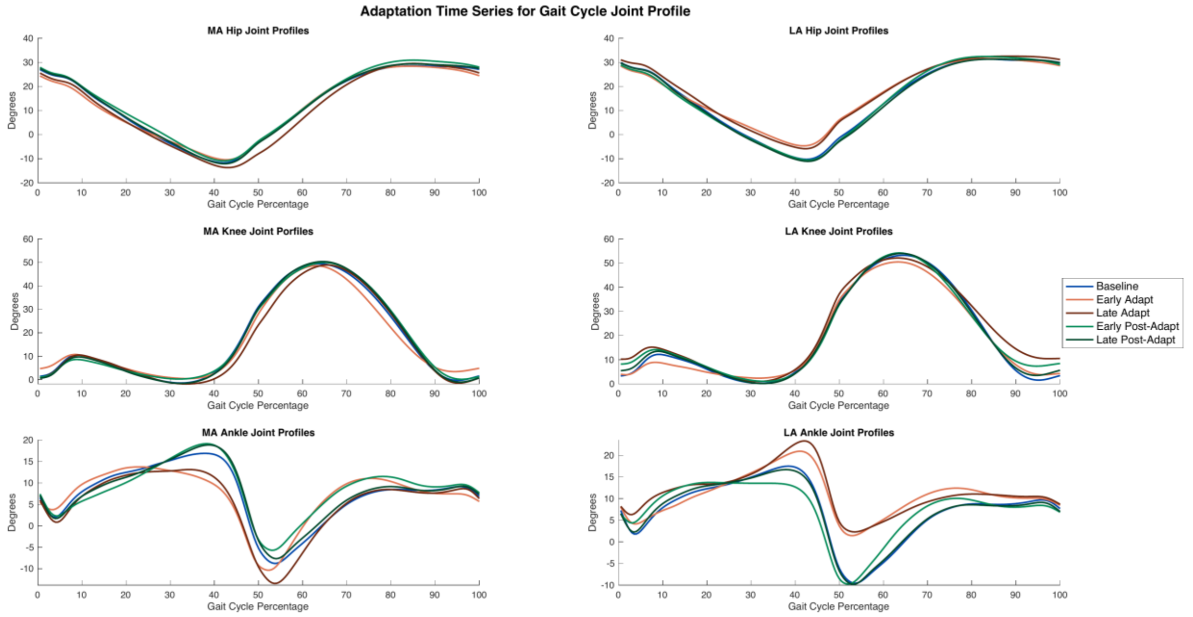

Figure S1. Joint profiles for the MA and LA hip, knee, and ankle for each timepoint of the split-belt adaptation paradigm. Joint profiles are averaged across all participants. Majority of change between timepoints occurred at the ankle joint, with less change occurring in the hip and knee joints.

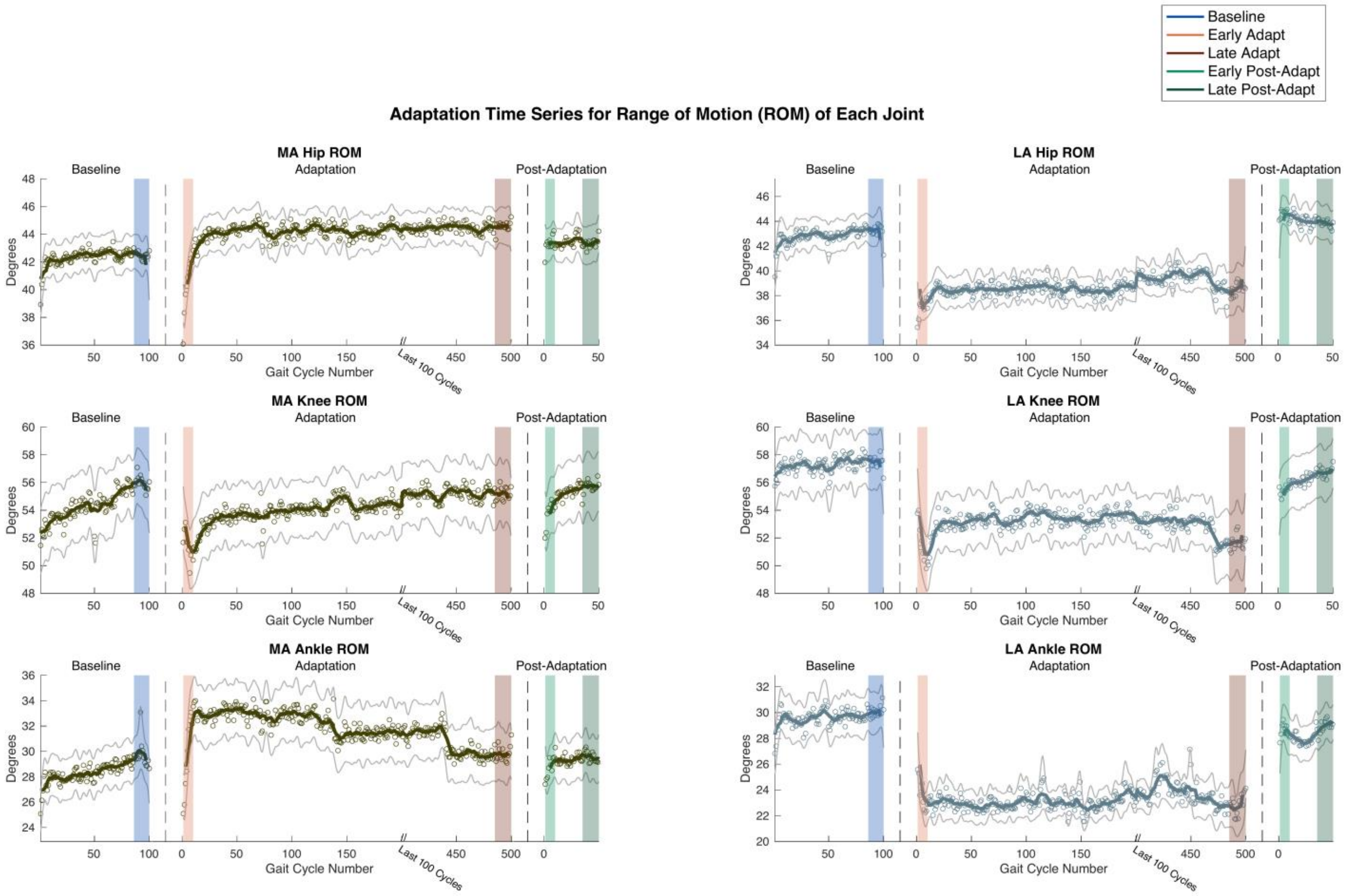

Figure S2. ROM adaptation for the MA and LA hip, knee, and ankle for Baseline, Adaptation, and Post-Adaptation. Light grey lines indicate standard error for each point across all participants. The

LA limb had immediate decreases in ROM in all joints from Baseline to Adaptation but demonstrated no adaptation.

Table S1. 2 x 5 RMANOVA pairwise results for each force variable. Following identification of a significant interaction effect pairwise comparisons between each timepoint were made using false discovery rate to correct for multiple comparisons.

Variable	Contrast	Interaction Effect (F)	Estimate (% BW)	Effect Size (d)
Propulsion Asymmetry[1]	Early Adapt – Baseline	38.78***	0.054***	1.40
	Late Adapt – Early Adapt		0.030***	0.56
	Early Post-Adapt – Baseline		-0.028***	0.79
MA Peak Propulsion Impulse[2]	Early Adapt – Baseline	11.51***	-0.12	0.075
	Late Adapt – Early Adapt		0.80***	0.47
	Early Post-Adapt – Baseline		-0.71***	0.49
LA Peak Propulsion Impulse[2]	Early Adapt – Baseline		-1.61***	1.19
	Late Adapt – Early Adapt		0.15	0.13
	Early Post-Adapt – Baseline		0.11	0.07
MA Peak Propulsion	Early Adapt – Baseline	12.09***	-0.022**	0.39
	Late Adapt – Early Adapt		0.048***	0.81
	Early Post-Adapt – Baseline		-0.026***	0.48
LA Peak Propulsion	Early Adapt – Baseline		-0.076***	1.61
	Late Adapt – Early Adapt		0.018**	0.48
	Early Post-Adapt – Baseline		0.0015	0.03
MA Peak Braking	Early Adapt – Baseline	8.12***	-0.078***	1.16
	Late Adapt – Early Adapt		0.11***	1.51
	Early Post-Adapt – Baseline		0.025*	0.25
LA Peak Braking	Early Adapt – Baseline		-0.039***	0.62
	Late Adapt – Early Adapt		-0.010	0.18
	Early Post-Adapt – Baseline		-0.067***	1.15
MA Peak Early Vertical GRF	Early Adapt – Baseline	4.92***	-0.10***	0.81
	Late Adapt – Early Adapt		0.054**	0.45
	Early Post-Adapt – Baseline		0.025	0.15
LA Peak Early Vertical GRF	Early Adapt – Baseline		0.0016	0.012
	Late Adapt – Early Adapt		0.032	0.26
	Early Post-Adapt – Baseline		-0.055***	0.51
MA Peak Late Vertical GRF	Early Adapt – Baseline	6.51***	-0.12***	1.22
	Late Adapt – Early Adapt		0.068**	0.72
	Early Post-Adapt – Baseline		-0.013	0.14
LA Peak Late Vertical GRF	Early Adapt – Baseline		-0.044**	0.60
	Late Adapt – Early Adapt		0.013	0.24
	Early Post-Adapt – Baseline		-0.083***	0.97

* = $p < 0.05$, ** = $p < 0.01$, *** = $p < 0.001$, 1. In the case of interlimb parameters, this is a main effect of timepoint rather than an interaction effect of limb x timepoint. 2. Units for impulse are %BW x %Stance.

Table S2. 2 x 5 RMANOVA pairwise results for each kinematic variable. Following identification of a significant interaction effect pairwise comparisons between each timepoint were made using false discovery rate to correct for multiple comparisons.

Variable	Contrast	Interaction Effect (F)	Estimate (degrees)	Effect Size (d)
Peak Dorsiflexion Asymmetry[1]	Early Adapt – Baseline	17.72***	-5.27***	0.97
	Late Adapt – Early Adapt		-2.98**	0.38
	Early Post-Adapt – Baseline		2.57**	0.55

MA Peak Dorsiflexion Time[2]	Early Adapt – Baseline	41.17***	-22.34***	1.64
	Late Adapt – Early Adapt		5.21	0.32
	Early Post-Adapt – Baseline		2.25	0.35
LA Peak Dorsiflexion Time[2]	Early Adapt – Baseline		8.86**	0.79
	Late Adapt – Early Adapt		1.48	0.19
	Early Post-Adapt – Baseline		-16.66***	0.94
MA Peak Hip Extension	Early Adapt – Baseline	3.97**	1.51**	0.24
	Late Adapt – Early Adapt		-2.97***	0.44
	Early Post-Adapt – Baseline		1.32	0.20
LA Peak Hip Extension	Early Adapt – Baseline		5.80***	0.86
	Late Adapt – Early Adapt		-1.31	0.20
	Early Post-Adapt – Baseline		-0.50	0.72
MA Knee Flexion[3]	Early Adapt – Baseline	0.30	-0.93	0.079
	Late Adapt – Early Adapt		0.35	0.042
	Early Post-Adapt – Baseline		-0.57	0.054
LA Knee Flexion[3]	Early Adapt – Baseline		-3.69	0.38
	Late Adapt – Early Adapt		2.05*	0.22
	Early Post-Adapt – Baseline		0.47	0.049
MA Peak Plantarflexion	Early Adapt – Baseline	17.10***	-2.23*	0.23
	Late Adapt – Early Adapt		-2.67*	0.52
	Early Post-Adapt – Baseline		2.82**	0.35
LA Peak Plantarflexion	Early Adapt – Baseline		9.92***	1.40
	Late Adapt – Early Adapt		1.54	0.26
	Early Post-Adapt – Baseline		0.29	0.04
MA Peak Dorsiflexion	Early Adapt – Baseline	13.90***	-1.87**	0.38
	Late Adapt – Early Adapt		-1.18	0.24
	Early Post-Adapt – Baseline		1.35	0.26
LA Peak Dorsiflexion	Early Adapt – Baseline		3.40***	0.71
	Late Adapt – Early Adapt		2.40*	0.38
	Early Post-Adapt – Baseline		-1.23	0.29

* = $p < 0.05$, ** = $p < 0.01$, *** = $p < 0.001$, 1. In the case of interlimb parameters, this is a main effect of timepoint rather than an interaction effect of limb x timepoint. 2. Units for peak dorsiflexion time are % gait cycle. 3. In cases of an insignificant interaction effect, we performed pairwise comparisons for demonstrative purposes but did not test for significance.
